# Commentary: Maintenance with hypomethylating agents after allogeneic stem cell transplantation in acute myeloid leukemia and myelodysplastic syndrome: A systematic review and meta-analysis

**DOI:** 10.3389/fmed.2022.1051526

**Published:** 2022-10-31

**Authors:** David Beauvais, Kévin-James Wattebled, Elodie Drumez, Ibrahim Yakoub-Agha

**Affiliations:** ^1^Univ. Lille, CHU Lille, Department of Hematology, Lille, France; ^2^Univ. Lille, CHU Lille, INSERM, Infinite, Lille, France; ^3^CHU Lille, Department of Biostatistics, Lille, France; ^4^Univ. Lille, CHU Lille, ULR 2694 - METRICS: Évaluation des technologies de santé et des pratiques médicales, Lille, France

**Keywords:** meta-analysis, immortal time bias, allogeneic hematogenetic stem cell transplantation, hypomethylating agent, acute myeloid leukemia, myelodysplastic syndromes

## Introduction

We read with great interest the study by Kungwankiattichai et al. entitled: “Maintenance With Hypomethylating Agents After Allogeneic Stem Cell Transplantation in Acute Myeloid Leukemia and Myelodysplastic Syndrome: A Systematic Review and Meta-Analysis” (1). Indeed, the value of maintenance therapy with hypomethylating agents (HMA) in the post-transplant setting has long been debated. In addition, 2022 European LeukemiaNet does not recommend subcutaneous azacytidine maintenance (2). In the current meta-analysis, Kungwankiattichai et al. reported a higher overall survival and relapse-free survival of the HMA maintenance group compared to the observation group. Moreover, the cumulative incidence of relapse and non-relapse mortality was significantly lower in those who received HMA. Therefore, the authors concluded that HMA maintenance after allogeneic hematopoietic cell transplantation (allo-HCT) was beneficial in patients with acute myeloid leukemia (AML) and myelodysplastic syndrome (MDS).

## Immortal time bias

As meta-analysis could be useful to pool individual studies to address a question, we believe that the meta-analysis in the context of post-transplant setting is still challenging. Such work may even amplify the bias or misinterpretation involved in many retrospective studies. Indeed, on one hand, patients who received HMA after transplant (at day+60 post-transplant for example) are only those who survived by day+60. On the other hand, patients who died prematurely are by default in the control group without HMA due to a well-known statistical bias called “immortal time bias” (3, 4). The elapsed time between allo-HCT and HMA initiation is an immortality period during which subjects who were candidate to receive HMA but died prematurely are counted as patients without HMA. Consequently, the immortal time bias generally tends to falsely attribute significant benefit to the study drug group.

The meta-analysis of Kungwankiattichai et al. consisted of 14 studies including 10 retrospective studies and 4 prospective studies. To avoid immortal time bias, two randomized studies were designed with a randomization occurring after transplant (between 42 and 100 days) (5, 6). Of note, Gao et al. observed reduced incidence of relapse with decitabine maintenance coupled with granulocyte colony-stimulating factor (G-CSF) while Oran et al. did not find any beneficial of 5-azacytidine (AZA) maintenance. From the outset, one may question the relevance of combining 2 studies using different drugs including one with additional G-CSF.

In reviewing the other studies included in the meta-analysis, some had not accounted the immortal time bias (7–11). All patients were included at time of transplantation leading to an overestimation of the efficacy of HMA. Moreover, while the studies account for confounding bias, the authors of the meta-analysis combined the outcomes of individual studies by pooling unadjusted risk ratio without accounting for confounding factors and without considering that the outcomes were censored events. In addition, some studies used prophylactic donor lymphocytes infusion (7, 12) or gemtuzumab ozaogamicin (8), which may further overestimate the effect of HMA. Finally, data recovery was probably an issue because some of studies were not published as regular article but as a letter or as a poster at conference (7) and indeed we were not able to find 3 analyzed studies (Ovechkina et al., Américo et al., Booth et al., for full references see Kungwankiattichai et al.).

## Discussion

For all these limitations, we believe that it is difficult to draw any firm conclusion based on Kungwankiattichai et al. There are ways to avoid immortality bias and every retrospective study should apply them. One is to avoid adding an immortal period by beginning follow-up of exposed and unexposed patients at the end of the identified immortal period (13). Alternatively, it is possible to compare 2 periods with a different treatment strategy in each period (e.g., all patients received treatment in period 1 and no patients received treatment in period 2). In addition, the use of a model that considers exposure as a time-dependent variable leads to a more potent effect. This method considers patients as unexposed from the beginning of the follow-up until the date on which they meet the criteria defining exposure and considers them as exposed after this date (14, 15). At last, a nested case-control design is a robust statistical method which has already demonstrated its ability in other areas (16).

We recently published an article of AZA as post-transplant maintenance in high-risk myeloid malignancies (17). In this retrospective study including 185 patients (65: AZA, 120: control group), the two groups were similar in terms of 2-year incidence of relapse, overall survival, and event-free survival. But before stating this result, we have been careful to consider the following points. First, we decided to exclude all patients who died, relapsed, or developed grade ≥2 acute graft-vs.-host disease before day+60, which avoided the immortal time bias. Second, we used “time-to-event” methods (Kaplan-Meier, Kalfbleisch and Prentice) to estimate survival and account for censored observations and competing risks or incidence. Third, we used multivariable analysis (multivariable Cox and Fine-Gray regression models) to avoid confusion bias and estimate the own effect of AZA.

In conclusion, meta-analyses are the highest level of evidence. But the best way to perform such analyses is to carry them out with accurate and complete data from the original studies. The flawed approach to data design and analysis, leading to an immortal time bias, may lead to a false conclusion, and generally favor the study treatment (14). Because of this limitation, we believe that to assess the benefit of HMA maintenance treatment after allograft, only a randomized study can allow a definitive conclusion.

## Author contributions

DB and K-JW analyzed the studies and wrote the initial version of the manuscript. ED identified the statistical issues. IY-A wrote the final version of the manuscript. All authors read and approved the final manuscript.

## Conflict of interest

The authors declare that the research was conducted in the absence of any commercial or financial relationships that could be construed as a potential conflict of interest.

## Publisher's note

All claims expressed in this article are solely those of the authors and do not necessarily represent those of their affiliated organizations, or those of the publisher, the editors and the reviewers. Any product that may be evaluated in this article, or claim that may be made by its manufacturer, is not guaranteed or endorsed by the publisher.
